# Lysosome sorting of β-glucocerebrosidase by LIMP-2 is targeted by the mannose 6-phosphate receptor

**DOI:** 10.1038/ncomms5321

**Published:** 2014-07-14

**Authors:** Yuguang Zhao, Jingshan Ren, Sergi Padilla-Parra, Elizabeth E. Fry, David I. Stuart

**Affiliations:** 1 Division of Structural Biology, University of Oxford, The Henry Wellcome Building for Genomic Medicine, Headington, Oxford OX3 7BN, UK; 2 These authors contributed equally to this work

## Abstract

The integral membrane protein LIMP-2 has been a paradigm for mannose 6-phosphate receptor (MPR) independent lysosomal targeting, binding to β-glucocerebrosidase (β-GCase) and directing it to the lysosome, before dissociating in the late-endosomal/lysosomal compartments. Here we report structural results illuminating how LIMP-2 binds and releases β-GCase according to changes in pH, via a histidine trigger, and suggesting that LIMP-2 localizes the ceramide portion of the substrate adjacent to the β-GCase catalytic site. Remarkably, we find that LIMP-2 bears P-Man_9_GlcNAc_2_ covalently attached to residue N325, and that it binds MPR, via mannose 6-phosphate, with a similar affinity to that observed between LIMP-2 and β-GCase. The binding sites for β-GCase and the MPR are functionally separate, so that a stable ternary complex can be formed. By fluorescence lifetime imaging microscopy, we also demonstrate that LIMP-2 interacts with MPR in living cells. These results revise the accepted view of LIMP-2–β-GCase lysosomal targeting.

## Abstract (web summary)

LIMP-2 is a membrane protein involved in β-glucocerebrosidase lysosomal targeting. Here Zhao *et al*. report structural and biochemical results showing how LIMP-2 interacts with β-glucocerebrosidase and the mannose 6-phosphate receptor, and propose a mechanism for LIMP-2-associated lysosomal targeting.

## Graphical abstract

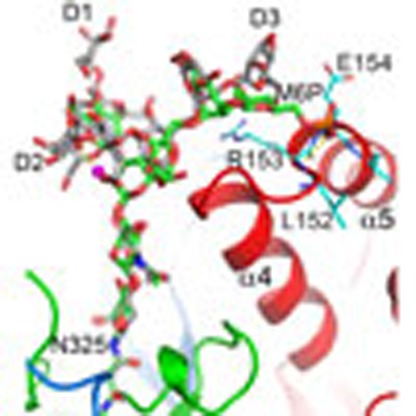

Lysosomal integral membrane protein 2 (LIMP-2) is encoded by gene *SCARB2*, and is also known as scavenger receptor class B member 2 (SCARB2; refs 1, 2). It has N- and C-terminal transmembrane helices (being a type III membrane protein) and a heavily glycosylated 400 residue luminal domain (bearing nine potential N-linked glycosylation sites)^3^. LIMP-2 has a major role in lysosomal and endosomal membrane organization, and LIMP-2 mutations cause several neurodegenerative and renal diseases, such as myoclonic epilepsy and nephrotic syndrome^4^,^5^. LIMP-2 is a member of the CD36 superfamily of scavenger receptors^6^,^7^, and has been identified as a receptor for Enterovirus 71 (EV71) and Coxsackievirus A16, which cause major epidemics of hand, foot-and-mouth disease in young children^8^. Structure determination of the lysosomal domain of LIMP-2 at pH 5.5 recently revealed a remarkable molecule harbouring a tunnel and homology modelling suggested a lipid transfer mechanism for the related molecules CD36 and SR-BI^9^. The most notable function of LIMP-2 is that it serves as a lysosomal sorting receptor for β-glucocerebrosidase (β-GCase)^10^, deficiency of which causes Gaucher disease, a lysosomal storage disease in which glucocerebrosides accumulate^11^. A short motif in the cytoplasmic tail of LIMP-2 has been thought to be solely responsible for lysosomal targeting^10^,^12^, making it a paradigm for mannose 6-phosphate (M6P) receptor (MPR)-independent lysosomal targetting. LIMP-2 binds to β-GCase in a pH-dependent manner, and dissociation occurs in acidic late endosomal/lysosomal compartments. Here, by determining a structure at a different pH, we are able to show the mechanism in atomic detail of pH-dependent release of β-GCase. The new structure also shows that LIMP-2 is modified by the post-translational addition of M6P, suggesting that it interacts with MPR. We show using biophysical methods and live cell microscopy that this interaction does occur and, by formation of a ternary complex of MPR, LIMP-2 and β-GCase provides a subcellular targeting mechanism.

## Results

### Structure determination

Human LIMP-2 luminal domain containing residues 28–431 was produced by stable expression in HEK293S (GnTI−) cells and crystallized in two space groups, *C*222_1_ and *C*2, at pH 6.5. X-ray diffraction data were collected at the Diamond Light Source, and structures of both crystal forms were determined using the molecular replacement method based on the model of LIMP-2 at lower pH^9^ and refined at 2.8 Å resolution. The final refined models have reasonable *R*-factors and very good stereochemistry. Details of protein expression, purification, crystallization and structure determination are given in Methods section and Table 1.

### Overall structure

**Figure 1:**
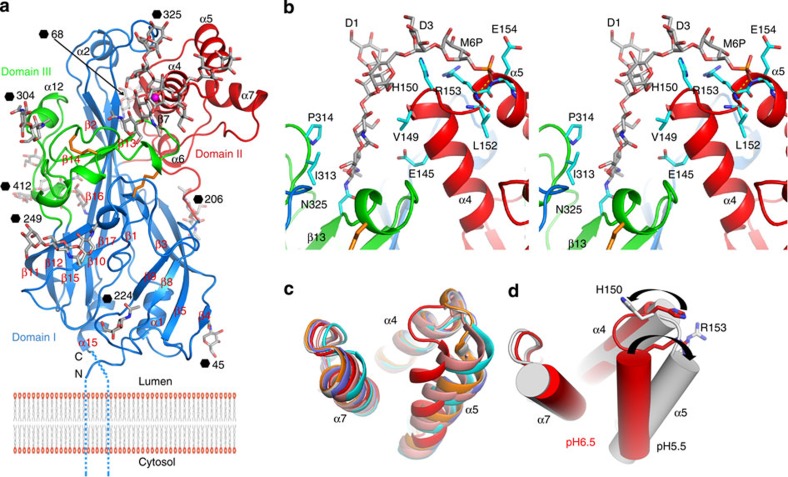
Overall structure of LIMP-2 and structural differences at pH 6.5 and 5.5. (**a**) Ribbon diagram showing the overall structure of LIMP-2 with domains I–III coloured in blue, red and green, respectively. Disulphides are drawn as orange sticks. Secondary structures are named as in Neculai *et al*.^9^ (**b**) Stereo view of the N-linked P-Man_9_GlcNAc_2_ (grey sticks) at N325 in space group *C*2, and its interactions with the protein (side chains as cyan sticks). (**c**) Structural differences in the domain II helical bundle at pH 6.5 (red) and those from six molecules at pH 5.5. (**d**) The switch in conformation of the helical bundle from pH 6.5 (red) to pH 5.5 (grey) is indicated by the black arrows.

**Figure 2:**
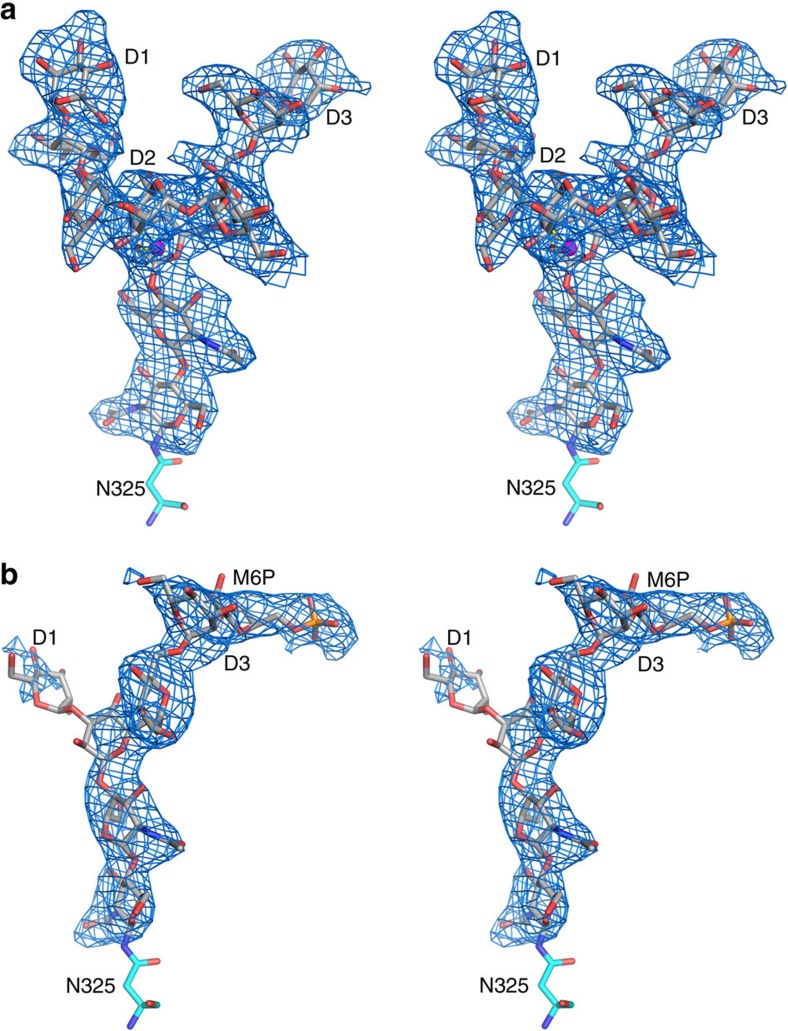
Simulated annealing omit *|F*_o_*-F*_c_*|* electron density map contoured at 2.5*σ*. (**a**) Stereo diagram showing the well-defined electron density for the N-linked Man_9_GlcNAc_2_ at N325 in space group *C*222_*1*_. A Na^+^ liganded by O2 of the β-mannose and O4 of the D2 mannose is shown as a magenta sphere. The phosphate at the D3 mannose is disordered. (**b**) The same glycosylation site in space group *C*2 showing the density for the M6P, but in this case residues in the D1 and D2 arms are disordered.

**Figure 3:**
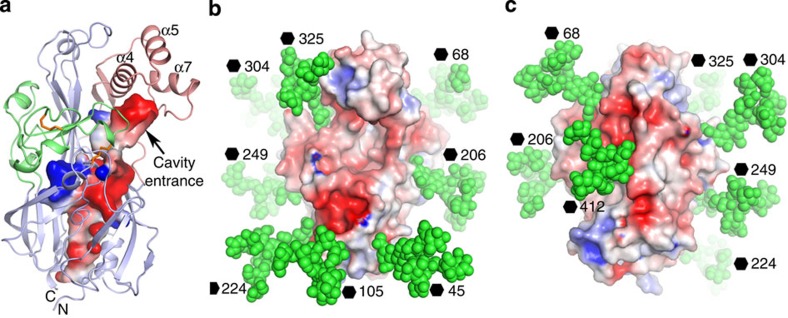
Structural features of LIMP-2. (**a**) Electrostatic surface contoured at±5 kT e^−1^ showing the tunnel and cavity in LIMP-2. The protein chain is shown as ribbons with its three domains coloured in pale blue, red and green, respectively. (**b**) Man_9_GlcNAc_2_ observed at N325 site is modelled at all nine glycosylation sites of LIMP-2 with atoms shown as green spheres on the electrostatic surface of the molecule to show glycan-free areas on the protein surface. (**c**) Approximately 180**°** rotation of **b** showing the back of the molecule.

Residues 38–429 of the bean-shaped molecule are visible, the structure fading out nine and two residues (at the N- and C-termini, respectively) from the terminal trans-membrane helices (Fig. 1a). The electron density maps for both space groups are of good quality with only 11 terminal residues disordered with 29 and 30 glycosidic units well ordered in the two space groups (Fig. 2 and Supplementary Table 1). Although sugars are seen on all nine potential sites, the electron density is too diffuse to permit modelling for the sugar attached to residue N105. The structure can be divided into three domains (Fig. 1a). Domain I, the core of the structure, comprises a 13-stranded antiparallel β-barrel wedged open at the membrane-proximal end by the insertion into the barrel of the β9–β10 loop. The narrower membrane-distal part of domain I (formed from the three longest strands: β1, β16 and β17) is flanked by two smaller domains, II and III. Domain II (residues 126–205), inserted between strands β5 and β8, and resting on the inner face of the three long strands of domain I, has two β-strands and four helices, three of which (α4, α5 and α7) form a bundle. Domain III (residues 297–365), inserted between β12 and β15, contains two loose coil regions separated by two short β-strands that pin the domain to β16 of the β-barrel. Remarkably, a large tunnel extends through the centre of domain I from the membrane proximal end to near the top of the molecule, where it opens into a large cavity at the base of domain II, the domain to which β-GCase attaches (Fig. 3a)^10^. Similar tunnels in the related molecules SR-BI and CD36 act as a route for lipid transport^9^, but the role in LIMP-2 has remained obscure^9^. However, the residues lining the tunnel are highly conserved from zebrafish to man, suggesting that the function is preserved.

### LIMP-2 possesses an N-linked P-Man_9_GlcNAc_2_

Not only does the electron density map show that all nine potential N-linked glycosylation sites in LIMP-2 bear sugars, but also that the density is sufficiently clear that at four positions five or more glycosides can be modelled. All these nine sites are conserved among different species apart from zebrafish, while the overall level of amino-acid identity is only ∼32% (ref. 9). The most surprising observation is that we can identify from the well-defined electron density that the sugar attached to N325 is P-Man_9_GlcNAc_2_ (Fig. 2), the first time this sugar, which bears a terminal M6P, has been seen in a protein structure. N325 is the last residue of β13 and sits at the bottom of a cleft formed by α4 and the α11-β13 loop (Fig. 1b). The first three saccharides of the glycan lie against α4 making mainly hydrophobic interactions on one side and ring-stacking interactions with a conserved *cis*-proline (P314) in the α11–β13 loop, on the other side. The region around the N325 glycosylation site is stabilized by two conserved disulphides, C312–C318 within the α11-β13 loop and C274–C329 that links the β13–β14 loop to the α9–β11 loop. In the *C222*_*1*_ crystal, the sugars are also stabilized by interaction with residues from a symmetry-related molecule that pack directly at the origin of the three arms of the glycan, forming a network of hydrogen bonds (Supplementary Figs 1 and 2), although this destabilizes the distal phosphate group. A well-defined ion (likely Na^+^) bridges the O2 atom of the β-mannose to O4 of the terminal D2 mannose (glycan nomenclature is defined in Supplementary Fig. 1a). The D3 arm of the glycan arches over the side chains of H150 and R153 that cap helix α4. The phosphate attached to O6 of the terminal D3 mannose is stabilized in the *C2* crystal form by capping the N terminus of helix α5 via hydrogen bonds to the amide groups of R153, E154 and I155 (Figs 1 and 2).

### P-Man_9_GlcNAc_2_ is positioned to bind MPR

**Figure 4:**
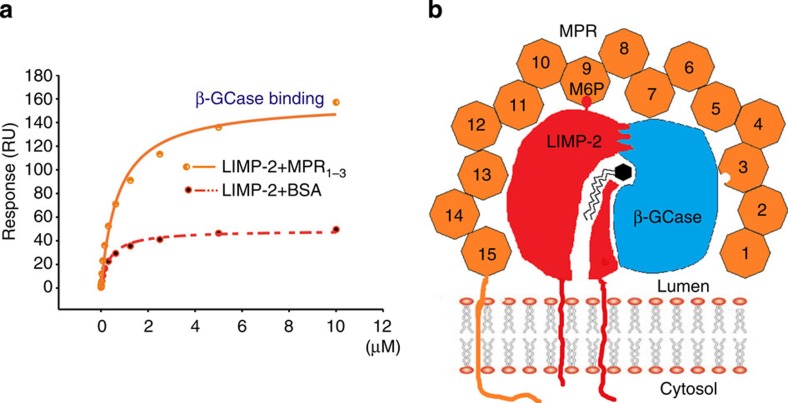
LIMP-2-mediated interactions. (**a**) SPR experiment showing that β-GCase binds to a preformed LIMP-2/CI-MPR complex to form a ternary complex, and also binds LIMP-2 alone (see also Fig. 5). β-GCase was biotinylated and attached to a CM5 chip, LIMP-2 mixed with MPR domains I–III in 1:1 ratio was then flowed over the chip. LIMP-2 mixed with BSA was used as a control. (**b**) A cartoon showing the complex of LIMP-2/β-GCase/CI-MPR. The orange octagons represent the 15 homologous domains of CI-MPR with its two M6P binding sites at domains III and IX. Which of the sites interacts with the M6P of LIMP-2 remains to be determined. The zig-zag lines with a hexagon head represent the glucocerebroside substrate of β-GCase. RU, resonance units.

Lysosomal enzymes are generally modified with a P-Man_9_GlcNAc_2_ that is recognized by the MPR for transport^13^. The LIMP-2 we used for structure determination was produced by stable expression in HEK293S cells (see Methods section). These cells are deficient in the enzyme β1,2-N-acetylglucosaminyltransferase I (GNTI) that is required to process complex sugars from Man_5_GlcNAc_2_ (ref. 14). We would therefore expect that most sugars will be of this type; however, the electron density at residue N325 demonstrates that this deficiency in the sugar processing is not a hindrance to proper P-Man_9_GlcNAc_2_ production. We presume that LIMP-2 therefore harbours a structural motif, which is known to be common to many soluble lysozymal enzymes, recognized by GlcNac-1 phosphotransferase^15^, the enzyme that phosphorylates the immature sugar. The presence of M6P suggested to us that MPR might attach to LIMP-2 in order to transport β-GCase to the lysosome in a hetero-trimeric complex (Fig. 4). In support of this, mutation of the residue N325 by which the M6P is attached to LIMP-2 abrogates transport to the lysosome^9^. We have confirmed this using confocal fluorescence microscopy (Supplementary Fig. 3), and have demonstrated that this is unlikely to be due to protein mis-folding, since soluble protein bearing the N325Q mutation could still be secreted (Supplementary Fig. 3c). If an MPR does bind it could either be the 46-kDa cation-dependent receptor that functions as a dimer, or the 300-kDa cation-independent (CI) receptor that contains 15 structural homology repeats with two M6P binding sites^16^, both are type I membrane proteins. Given the large distance from the M6P to the membrane in LIMP-2 (∼70 Å based on the structure shown in Fig. 1a), the longer CI-MPR would be the most likely co-transporter (Fig. 4b).

### LIMP-2 binds CI-MPR via M6P and simultaneously binds β-GCase

**Figure 5:**
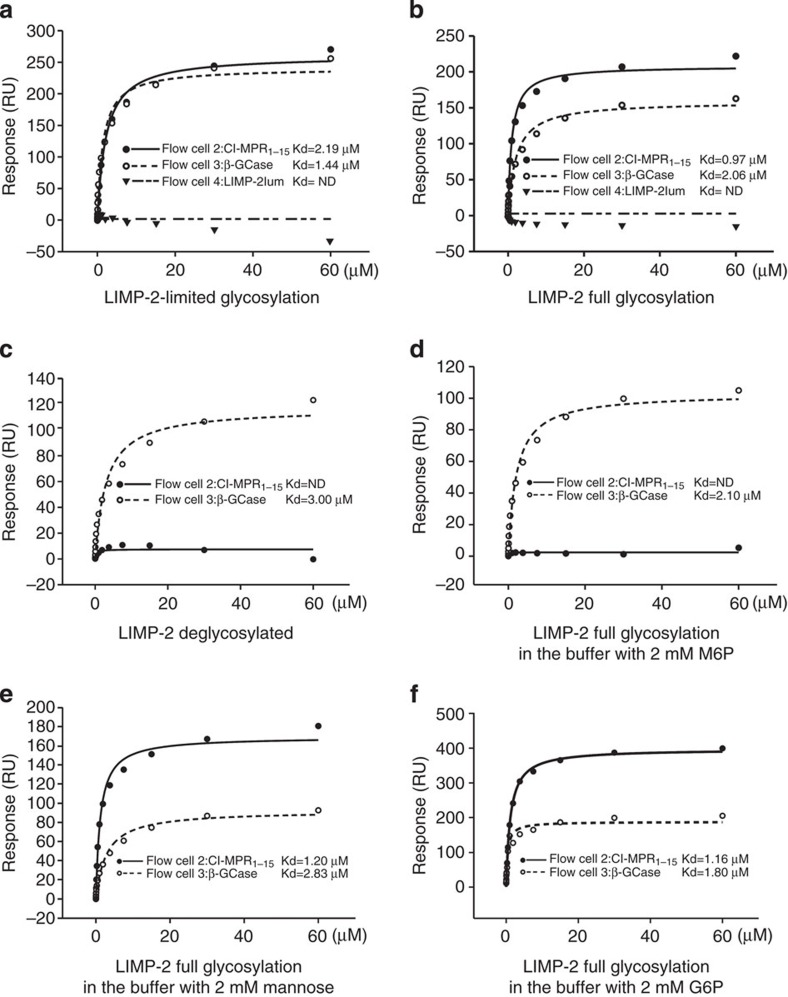
SPR experiments for the interaction of LIMP-2 with CI-MPR and β-GCase. Biotinylated human CI-MPR domains I–XV and β-GCase were immobilized in flow cells 2 and 3 of a CM5 chip, respectively. LIMP-2 in different glycosylation status and in different sugar containing buffer was used as analyte. (**a**) LIMP-2 produced from HEK293S (GNTI−) cells, with limited glycosylation. (**b**) LIMP-2 produced from HEK293T cells, with full glycosylation. (**c**) LIMP-2 produced from HEK293S (GNTI−) cells and deglycosylated with endoglycosidase F1. (**d**) Fully glycosylated LIMP-2 in buffer containing 2 mM M6P. (**e**) Fully glycosylated LIMP-2 in buffer containing 2 mM mannose. (**f**) Fully glycosylated LIMP-2 in buffer containing 2 mM G6P. ND, not detectable; RU, resonance units.

To establish if LIMP-2 and CI-MPR do indeed interact, we performed surface plasmon resonance (SPR) analyses (see Methods section). Three ligands were immobilized in a CM5 chip in parallel, with biotinylated CI-MPR domains I–XV, biotinylated β-GCase and biotinylated LIMP-2_lum_ attached to separate flow cells. Soluble LIMP-2 was then flowed over all cells. The first experiment used LIMP-2 produced from HEK293S (GNTI−) cells, the same material used for crystallization, which therefore had limited processing of the sugars. This version of LIMP-2 bound tightly to both CI-MPR (Kd 2.2 μM) and β-GCase (Kd 1.4 μM), but not to LIMP-2 itself (Fig. 5a). When fully glycosylated LIMP-2 produced from HEK293T cells was used, an even higher affinity of Kd 0.97 μM was achieved for CI-MPR (Fig. 5b). To test the glycosylation dependence of binding, we used endoglycosidase F1 to trim the glycans to a single N-acetylglucosamine. The deglycosylated LIMP-2 lost its ability to bind CI-MPR, whereas it retained its ability to bind β-GCase (Fig. 5c). To assess the specificity for M6P, we used M6P as a chemical competitor. In the presence of 2 mM M6P, LIMP-2 could not bind CI-MPR while binding to β-GCase was not affected (Fig. 5d). We then competed simply with mannose to test the requirement for the 6-phosphate group. Mannose (2 mM) did not affect LIMP-2 binding to CI-MPR (Fig. 5e). Finally, we tested with glucose-6-phosphate (G6P) to confirm that the binding is M6P specific. G6P (2 mM) did not affect LIMP-2 binding to CI-MPR (Fig. 5f). We also tested LIMP-2 produced from HEK293S GNTI− cells for CI-MPR binding in the presence of the above sugars (Supplementary Fig. 4) and confirmed that LIMP-2 with limited glycosylation behaves similarly to fully glycosylated LIMP-2. Those data clearly demonstrate that LIMP-2 binds CI-MPR in a M6P-dependent manner. To test if β-GCase, LIMP-2 and MPR form a hetero-trimeric complex, we attached biotinylated β-GCase to a CM5 chip. LIMP-2 mixed with an equal amount (1:1 molecular ratio) of CI-MPR (domain I–III, with Rodopsin 1D4 tag, produced from HEK293S GNTI− cells, see Methods section), or same amount of LIMP-2 mixed with an equal amount of BSA, was flowed over the same chip. The results show that the LIMP-2/MPR has a much higher response than LIMP-2/BSA, demonstrating the presence of the hetero-trimeric β-GCase/LIMP-2/MPR complex (Fig. 4).

### Detection of LIMP-2 and CI-MPR interaction in living cells

**Figure 6:**
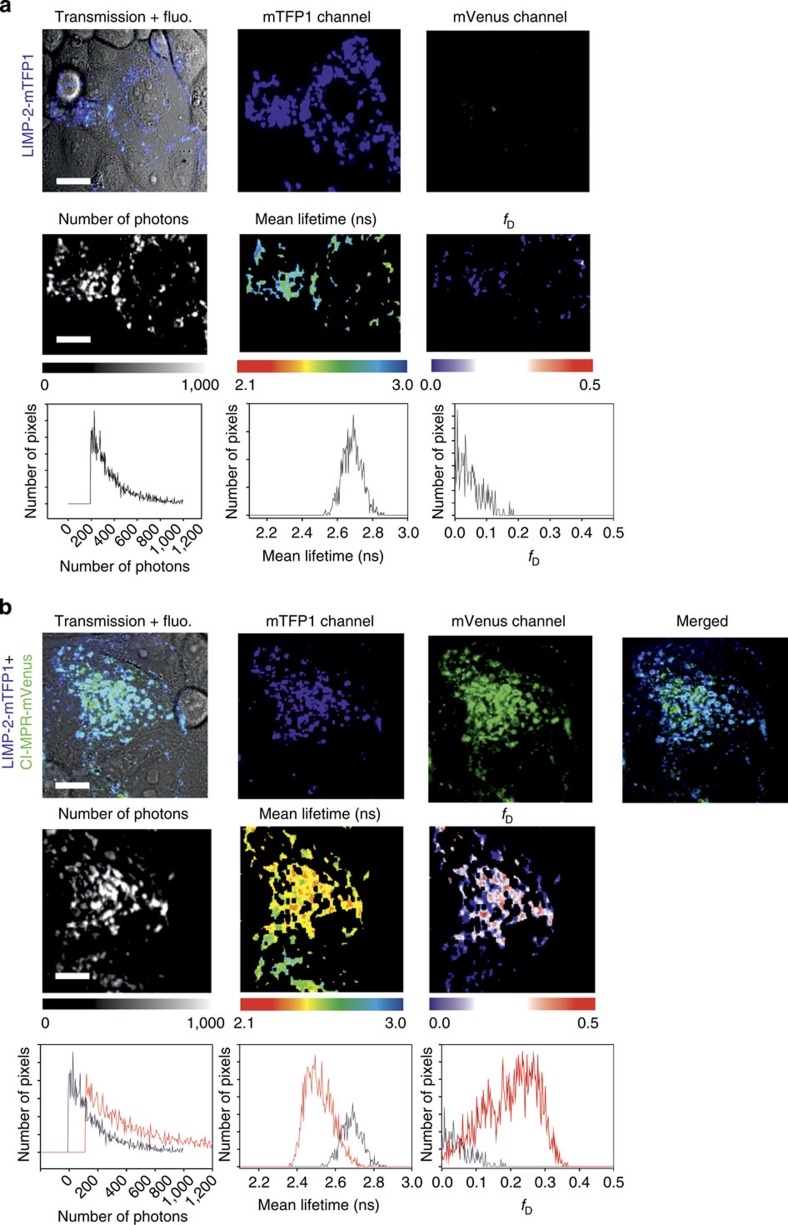
FLIM-FRET between LIMP-2 and CI-MPR. (**a**) COS 7 cells expressing LIMP-2-mTFP1 alone (first row) show a punctate appearance (second panel from the left). The average number of acquired photons after applying a 2 × 2 spatial binning is 413 photons, the average mean lifetime (<*τ*>) for this sample is 2.68 ns, and finally the average *f*_D_ is 5% (note in this and all subsequent panels, *f*_D_ corresponds to the fraction of LIMP-2 directly interacting with an acceptor). (**b**) COS-7 cells co-expressing LIMP-2-mTFP1 and CI-MPR-mVenus also show a punctate appearance and a high degree of co-localization (first row). The average number of photons after applying a 2 × 2 binning filter is ∼600, the average mean lifetime (<*τ*>) is 2.52 ns, which indicates FRET, the mean *f*_D_ for this sample is 28%. The average lifetime diminution is calculated in some pixels to be down to 2.32 ns (red pixels, second row of micrographs, second panel), which corresponds to ∼35% *f*_D_, (red pixels, second row of micrographs, third panel), that is, a substantial proportion of the LIMP-2 is involved in a direct interaction. fluo., fluorescent channel. Scale bar, 5 μm.

**Figure 7:**
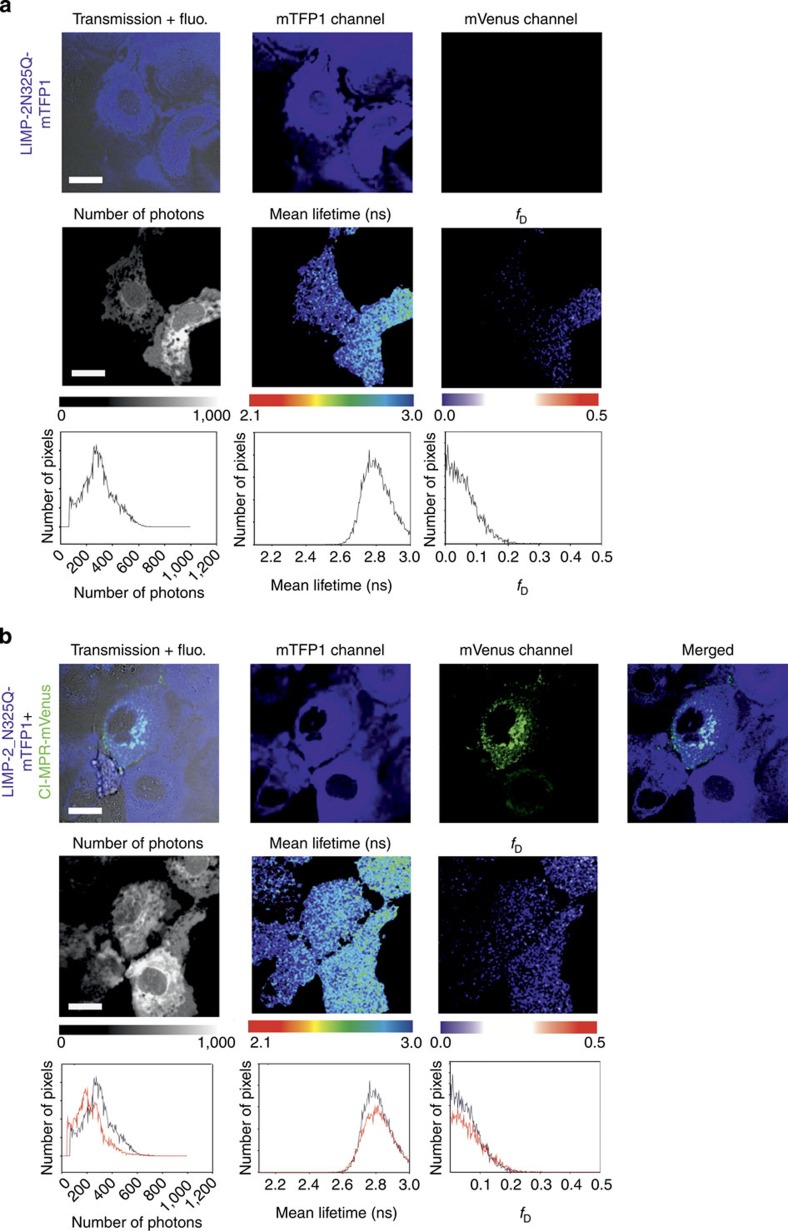
LIMP-2-N325Q mutant and CI-MPR show no detectable FRET. (**a**) COS-7 cells expressing LIMP-2-N325Q-mTFP1 shows a diffuse pattern (first row). The average number of photons after applying a 2 × 2 spatial binning is ∼300, the average mean lifetime is found to be 2.77 ns and the corresponding *f*_D_ of 6%, that is, essentially no interaction, see Fig. 6 and main text (second row). (**b**) COS 7 cells co-expressing LIMP-2-N325Q-mTFP1 and CI-MPR-mVenus show a diffuse pattern for LIMP-2-N325Q-mTFP1 (blue, second panel from the left) and punctate pattern for CI-MPR-Venus (third panel from the left). The FLIM image (second row, second panel) does not show a significant mean lifetime diminution (<*τ*>=2.77 ns), which is not significantly different from the control (LIMP-2-N325Q-mTFP1 alone), and hence no FRET is detected. Scale bar, 5 μm.

**Figure 8:**
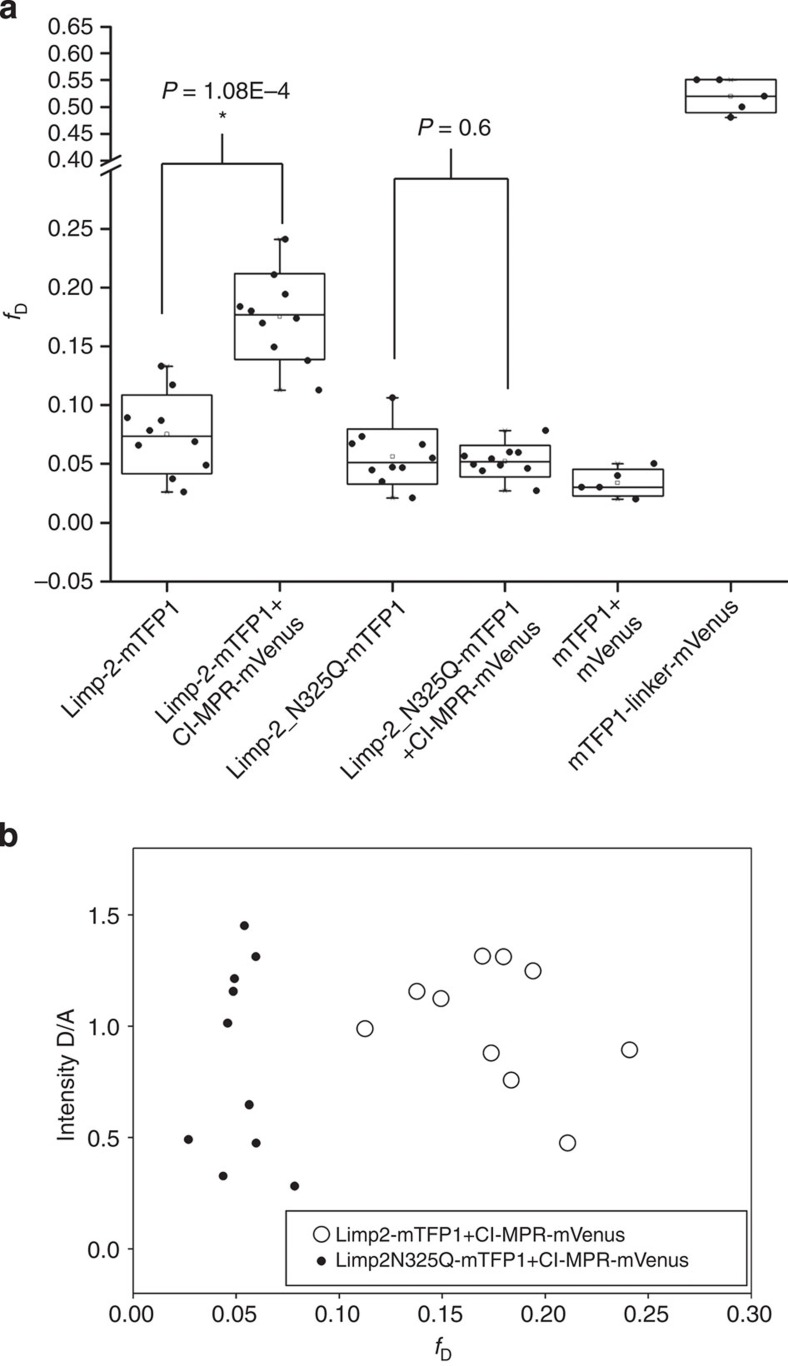
Statistic analysis of the FLIM-FRET from LIMP-2 and CI-MPR. (**a**) A box plot shows the mean (the box middle horizontal line), the s.d. (top and bottom of the box), the maximal and minimal values (whiskers) of *f*_D_ distribution for LIMP-2-mTFP1 expressed alone (*f*_D_=0.07±0.03, *n*=10) and LIMP-2-mTFP1 with CI-MPR-mVenus (*f*_D_=0.17±0.03, *n*=10 from three independent experiments). The *f*_D_ of the later is statistically significant compared with LIMP-2-mTFP1 alone (*P*=1.8 × 10^−4^). The corresponding *f*_D_ values from LIMP-2-N325Q-mTFP1 expressed alone (0.05±0.02, *n*=10) and LIMP-2-N325Q-mTFP1 with CI-MPR-mVenus (0.05±0.01) show no statistical difference (*P*=0.6). The fluorescent protein only donor (mTFP1) co-expressed with the acceptor (mVenus) is used as negative control while the donor (mTFP1) linked the acceptor (mVenus) with a flexible polypeptide chain of 13 amino acids is used as a positive control. The values were calculated using Sigma Plot (Student’s *t*-test). (**b**) Scatter plot showing the distribution of averaged mean *f*_D_ values against their corresponding donor and acceptor intensity ratio. A random distribution in both cases shows *f*_D_ values are independent of acceptor intensity.

Since soluble LIMP-2 and CI-MPR bind specifically *in vitro*, we tested the interaction of the full-length membrane proteins in living cells by fluorescence lifetime imaging to detect Förster resonance energy transfer (FLIM-FRET). FLIM-FRET provides direct information about protein–protein interactions provided that appropriately labelled donor and acceptor molecules are positioned within ∼10 nm with the right orientation (this proximity shortens the donor fluorescence lifetime due to FRET)^17^. A monomeric teal fluorescent protein 1 (mTFP1, a FRET donor)^18^ was attached to LIMP-2 and mVenus (a FRET acceptor)^19^ was attached to CI-MPR (see Methods section). First, we examined, in COS 7 cells, subcellular localization of fluorescently labelled LIMP-2 and CI-MPR, with mouse Ras-related protein Rab5a-mCherry as marker for early endosomes^20^. The punctate co-localization of LIMP-2 with Rab5a-mCherry and LIMP-2 with CI-MPR is evident (Supplementary Fig. 3). When COS 7 cells co-expressed (via co-transfection) both LIMP2-mTFP1 and CI-MPR-mVenus fusion proteins, the average diminution of the donor mean lifetime (<*τ*>) was 2.52±0.03 ns (*n*=11), significantly shorter (Student’s *t*-test, *P*=6.61 × 10^−6^) than the donor alone (<*τ*>) 2.65±0.04 ns (*n*=12), indicating FRET between LIMP-2 and CI-MPR. A representative experiment is shown in Fig. 6. In this particular example, the average lifetime diminution in some pixels was calculated to be little as 2.32 ns. We have also calculated the fraction of interacting donor (*f*_D_) to quantify the extent of this interaction^21^,^22^. Indeed, *f*_D_ is an interesting parameter to characterize protein–protein interactions in living cells rather than the true FRET efficiency (*E*) that defines the relative orientation of the donor relative to the acceptor^17^. In Fig. 6b, the average *f*_D_ coming from all pixels of the image was ∼0.25. Observe that we also calculated the error for the cell expressing LIMP-2-mTFP1 alone (∼0.05). However, when COS 7 cells were co-transfected with LIMP-2-N325Q-mTFP1 and CI-MPR-mVenus, the distribution of LIMP-2 within the cells became diffuse, whereas CI-MPR maintained the punctate pattern, and FLIM imaging no longer showed a significant mean lifetime diminution (<*τ*>=2.77 ns) as compared with LIMP-2-N325Q-mTFP1 expressed alone (<*τ*>=2.78 ns) The corresponding *f*_D_ here are really close to 0 (0.05 in both cases), and show the absence of interaction between LIMP-2-N325Q-mTFP1 and CI-MPR-mVenus (Fig. 7). When calculating these parameters for a population of cells (Fig. 8a), we found a mean of the average *f*_D_ of 0.17±0.03 for LIMP-2-mTFP1 and CI-MPR-mVenus interaction (*n*=10 from three independent experiments), and a mean of the average *f*_D_ of 0.05±0.01 for LIMP-2-N325Q-mTFP1 and CI-MPR-mVenus (*n*=10); this *f*_D_ corresponds to the error coming from the technique since a similar error was found for both LIMP-2-N325Q-mTFP1 and LIMP-mTFP1 expressed alone (Fig. 8a). A *t*-test between the groups showed a statistically significant difference only for the difference between LIMP-2-mTFP1 alone and LIMP-2-mTFP1 in the presence of MPR-mVenus (*P*=1.08 × 10^−4^). Importantly, when plotting the intensity ratio between the donor and the acceptor expression against the corresponding *f*_D_, we found a random distribution, confirming that the level of acceptor expression did not affect our results (Fig. 8b). These data demonstrate the *in vivo* interaction between LIMP-2 and CI-MPR, and suggest that the single N325Q mutation, which ablates the M6P attached to this residue, abolishes this interaction. We have shown above that this mutation does not prevent the secretion of the soluble version of this mutant, indicating correct folding.

### Potential binding site for β-GCase

In order to highlight the possible β-GCase and CI-MPR binding areas of LIMP-2, we have modelled all N-linked glycans of LIMP-2 as Man_9_GlcNAc_2_ (as a crude model for complex sugars). A glycan-free belt remains, traversing from the front, over the top, to the back of the molecule (Fig. 3). Since extra glycan in an H363N mutant or lack of glycan in an N68Q mutant both impair LIMP-2 trafficking to the lysosome^9^,^23^, and the former does not affect β-GCase binding, it is likely that MPR interacts with the back, and β-GCase binds to the front of LIMP-2 (Fig. 3). This provides a plausible explanation for the role of the tunnel in LIMP-2. In CD36 and SR-BI, this is used for lipid delivery, and if β-GCase binds with its active site facing the cavity of LIMP-2 then it could accommodate the long lipidic portion of the glucosylceramide substrate of β-GCase^24^. It is even possible that in the endosome, glucosylceramide is delivered from the inner leaflet of the membrane through the tunnel to the active site of β-GCase (Fig. 4b). This would imply that LIMP-2 acts as a regulatory subunit of β-GCase. We note, however, that it is also possible that the tunnel might harbour phosphatidylinositol or a derivative, as it has been established that phosphatidylinositol 4 kinases control lyososomal delivery of the LIMP-2/β-GCase complex^25^.

### Conformational changes in LIMP-2 mirror pH dependency of β-GCase binding

In the late endosome/lysosome β-GCase disassociates with the drop in pH, and associates instead with saposin C, which appears to increase the activity of the enzyme^26^. To understand this pH-dependent dissociation, we compared our pH 6.5 structures with the six independent molecules of the crystallographic structure at pH 5.5 (ref. 9). Although our two structures at pH 6.5 are almost identical to each other (root mean squared deviation=0.3 Å for all Cα atoms), they differ significantly from the pH 5.5 structures (root mean squared deviation=0.5–0.6 Å), with the largest difference occurring in the helical bundle of domain II, which includes exactly the region (residues 150–167) identified as the β-GCase binding site (Fig. 1c)^10^. This region shows pH-dependent changes in flexibility as well as conformation. At pH 5.5, the α4–α5 region shows considerable flexibility, the two-residue linker between α4 and α5 adopting two major conformations, three residues out of step with each other. In contrast, at pH 6.5, the helical bundle is very well defined and similar in both space groups. At higher pH, the α4–α5 linker (residues 151–152) folds towards α7, such that α5 tilts about 20**°** towards α4 and α7, making the bundle more compact and further stabilized by the C-terminal cap of α4 formed by His150 and Arg153 (Fig. 1b,d). At pH 6.5, residues 152–163 constitute α5, whereas at lower pH the helix starts either at 149 or 152 (Fig. 1c). Our structure, determined at pH 6.5, similar to that of the endoplasmic reticulum (ER) and endosome, is likely to be competent to bind β-GCase.

### Histidine 150 acts as a pH sensor

**Figure 9:**
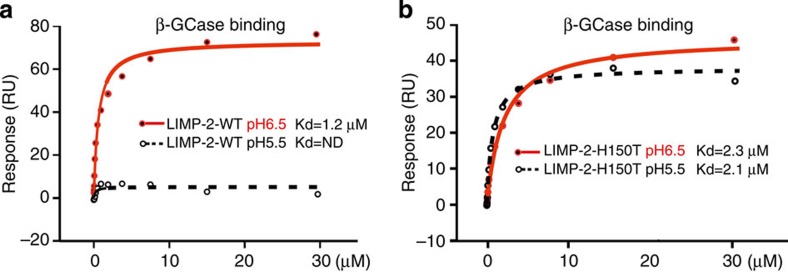
Interactions of β-GCase with wild-type and H150T mutant LIMP-2. SPR experiments showing binding of β-GCase to (**a**) wild-type LIMP-2 and (**b**) H150T mutant LIMP-2 at pH 5.5 and pH 6.5. Biotinylated β-GCase was immobilized on a CM5 chip. Wild-type and H150T mutant LIMP-2 were used as analyte in phosphate citrate buffer. ND, not detectable; RU, resonance units.

H171 has been proposed to act as a pH sensor^27^, however, it is located on β7 at the base of domain II (Supplementary Fig. 5), hydrogen bonds to E175 (3.2 Å) and does not undergo noticeable conformational change with pH. In contrast, H150 caps α4 at higher pH and seems more likely to act as the trigger for the markedly altered conformation of α4 and α5. At lower pH, H150 and R153 move away, decapping the α4 C terminus and triggering a cascade of structural change in the helical bundle that leads to the dissociation of β-GCase from LIMP-2. To establish if H150 is a pH sensor, we performed SPR experiments of β-GCase binding to H150T mutant LIMP-2 and used wild-type LIMP-2 as a control. The experiments clearly show that wild-type LIMP-2 binds β-GCase at pH 6.5, but not at pH 5.5, whereas the H150T mutant LIMP-2 binds β-GCase at both pH 6.5 and 5.5 (Fig. 9). The results suggest that the H150T mutant is locked in the β-GCase binding conformation seen in the higher pH structure and unable to switch conformation at lower pH, demonstrating that H150 is indeed a pH sensor.

## Discussion

In summary, our structures illuminate several aspects of the mechanism of β-GCase transport, indeed the location of a putative lipid transport channel suggests that LIMP-2 may act not only as a transporter but also as a regulatory subunit, analogous to the activity-enhancing saposin C that binds β-GCase following its release from LIMP-2 (ref. 26). In addition, LIMP-2 is a receptor for EV71 and Coxsackievirus A16 (refs 28, 29, 30), and residues 144–151, the C-terminal half of α4, are supposed to interact directly with the virus^28^,^29^. Since these residues are partially sheltered by a P-Man_9_GlcNAc_2_ attached to residue N325, glycan may serve as an attachment point for EV71 and CAV16 infection.

Following the observation of a P-Man_9_GlcNA_2_ moiety attached to LIMP-2, we demonstrated that LIMP-2 binds CI-MPR via M6P, the same mechanism used for classical lysosomal hydrolase transport. It is conceivable that further phosphorylated sugar may be attached elsewhere on the molecule, although we see no evidence for it. Although the affinity between LIMP-2 and CI-MPR is lower than that reported between a soluble lysosomal enzyme, β-glucuronidase and CI-MPR^31^, we have shown by SPR that the affinity of LIMP-2 for CI-MPR is similar to its affinity for β-GCase, and that these three proteins form hetero-trimeric complexes *in vitro*. It seems that MPR contributes indirectly to lysosomal targeting of β-GCase by binding the LIMP-2 component of the β-GCase/LIMP-2 complex. This is corroborated by FLIM-FRET results, demonstrating that LIMP-2 and CI-MPR are in close proximity in cells, while this co-localization is abolished for the mutant LIMP-2 lacking the M6P. This strongly suggests a direct interaction of the M6P with CI-MPR, however, it is conceivable that the asparagine mutation has an indirect effect. We note that while MPRs are essential in sorting a repertoire of over 60 different soluble acid hydrolases, this is only the second example of an MPR binding a membrane protein (CD26 binds CI-MPR, contributing to T-cell activation^32^). Our results contrast with the report that β-GCase targeting is MPR independent^10^, which found that β-GCase targeting is maintained in the presence of a mutation to GlcNac-1 phosphotransferase that traps the enzyme in the endoplasmic reticulum and abolishes the lysosomal targeting of soluble enzymes^33^. It may be that the mechanism of sorting is more complex and involves both MPR-dependent and MPR-independent processes.

Our results have also shown that the pH-dependent binding of β-GCase to LIMP-2 is related to conformational changes in the domain II helical bundle. We propose that H150, which caps the C terminus of α4, acts as a pH sensor that is in contrast to a previous report that H171 may function as a critical pH sensor^27^. We demonstrate that mutation of H150 to a threonine locks LIMP-2 in the high pH conformation, which binds β-GCase at both pH 6.5 and 5.5. It seems likely, therefore, that protonation of H150 at lower pH, for example, in the late endosome/lysosome environment, triggers conformational changes in the helical bundle and release of β-GCase from LIMP-2. However, since H150 is not conserved, it is possible that the mechanism is different in other species.

## Methods

### Protein production

Protein production used a variant on our standard mammalian production system^34^, modified for the stable expression of a secreted product. All the PCR amplifications were carried out with KOD hot start DNA polymerase (EMD Millipore) according to manufacturer’s recommendation (primers are listed in the Supplementary Table 2). Human LIMP-2 (UniProtKB/Swiss-Prot Q14108) luminal domain (residues V28–T431) was PCR amplified from IMAGE clone 3872778 (BioScience) and cloned into a newly made in-house stable cell line vector pNeoSec (Supplementary Fig. 6). HEK293S GnTI(−) cells were co-transfected with a pNeoSec-LIMP-2 and a PhiC31 integrase expression vector (pCB92/pgk-φC31; ref. 35). The polyclonal population resulting from G418 (1 mg ml^−1^) selection was cultured in Hyperflasks (Corning) in a CompacT SelecT-automated cell culture system (TAP Biosystems)^34^. The conditioned medium was dialysed and protein was purified with Talon Co^2+^ affinity resin (Clontech), and polished on a Superdex 200 16/60 column, eluted in 10 mM HEPES, pH 7.4, and 150 mM NaCl. To produce fully glycosylated LIMP-2, the same construct was transiently transfected into HEK293T (ATCC CRL-11268) cells and protein purified in the same way. CI-MPR (UniProtKB/Swiss-Prot P11717) domains I–III (residue Q41-L489) was cloned into pURD vector in frame with a Rhodopsin 1D4 tag^36^. A stable HEK293S GNTI− cell line was generated by puromycin selection, and protein was purified as reported previously^36^.

### Crystallization

LIMP-2 produced in HEK293S (GNTI−) cells was concentrated to 4 mg ml^−1^, and crystallization screening was carried out using the sitting-drop vapour diffusion method in 96-well plates^37^. Images were taken using a TAP Biosystems storage vault^38^ at 21 °C. Clusters of needles appeared in 20% (w/v) polyethylene glycol 3350, 0.1 M Bis-Tris propane, pH 6.5, and 0.2 M sodium acetate. Crystals were then crushed and cross-seeded into a set of crystallization screen conditions. The best diffracting crystals were obtained in 30% (w/v) polyethylene glycol 8000, 0.2 M ammonium sulphate and 0.1 M sodium cacodylate at pH 6.5.

### Data collection and structure determination

Crystals were flash frozen by immersion in a reservoir solution supplemented with 25% (v/v) glycerol followed by transfer to liquid nitrogen, and kept at −173 °C during X-ray data collection at I24, Diamond Light Source. Data images (exposure time 0.1 s with 30% beam transmission) of 0.2**°** rotation were recorded on a PILATUS 6M detector, at a wavelength of 0.9686 Å. Data images were indexed and integrated with HKL2000, and reflections were merged with SCALEPACK^39^. The two crystal forms diffracted to 2.8 Å resolution, with space groups of *C*222_1_ and *C*2. Structure determination by molecular replacement with a LIMP-2 search model (PDB ID 4F7B)^9^ used MOLREP^40^. Structure refinement used REFMAC^41^ and model rebuilding COOT^42^. Table 1 shows data collection and refinement statistics. Figures were prepared using PyMOL^43^.

### SPR equilibrium binding studies

Human β-GCase residues (40A-536R), human CI-MPR domains I–XV (residues 41Q-G2293) and human LIMP-2 luminal domain (residue V28-T431) were cloned into pHL-Avitag3 (ref. 44) and *in vivo* biotinylated by co-transfection with a BirA-ER plasmid^45^ into HEK293T cells. About 1,000 resonance units of each of the biotinylated proteins were immobilized on a CM5 sensor chip (GE Healthcare Life Sciences) to which streptavidin had been covalently coupled. We used a Biocore T100 machine (GE Healthcare) at 25 °C with a running buffer comprising 10 mM HEPES, pH 7.5, 150 mM NaCl and 0.005% Tween 20. The response was plotted versus the concentration of the analytes and fitted by nonlinear regression to a one-site saturation binding model (Sigma Plot, Systat software, Inc. San Jose, CA). For inhibition experiments, 2 mM M6P, 2 mM G6P or 2 mM mannose was included in the above running buffer. For pH-dependent binding, phosphate citrate buffer at either pH 6.5 or 5.5 with 150 mM NaCl and 0.005% Tween 20 was used.

### FLIM-FRET

Full-length human CI-MPR (residues Q41-I2491) was tagged at its C-terminal end in-frame with mVenus^19^ in a pHL-Sec-based vector^44^. Full-length human LIMP-2 (residues M1-T478) was tagged at its C-terminal end with mTFP-1 (ref. 18), and mouse Rab5a-mCherry was obtained from Addgene^46^ deposited by Christien Merrifield. The LIMP-2 N325Q mutation was made by a two-step overlapping PCR strategy and tagged with mTFP1 in the same way as the wild-type protein. COS 7 cells transiently co-expressing LIMP-2-mTFP1, CI-MPR-mVenus and Rab5a-mCherry, LIMP-2-mTFP1 and CI-MPR-mVenus, LIMP-2-N325Q-mTFP1 and CI-MPR-Venus or LIMP-2-mTFP1 (donor only, negative control) were grown to 60–70% confluence on glass-bottom 35 mm Petri dishes (Mattek) and imaged in phenol red-free medium. Multicolour images were acquired using a Leica SP8-X-SMD confocal microscope (Leica Microsystems, Manheim, Germany) with a × 63/1.4 numerical aperture oil immersion objective. LIMP-2-mTFP1 and CI-MPR-mVenus were excited with a 440-nm laser and a white light laser (WLL) tuned to 514 nm, respectively. Rab5a-mCherry was excited with the WLL tuned to 594 nm. Fluorescence emission for mTFP1 and mVenus-labelled proteins was detected with two HyD detectors capable of photon counting; the mCherry fluorescence was detected using a cooled photomultiplier tube. The emission windows for the fluorescent proteins utilized were selected as follows: mTFP1 (460–500 nm), mVenus (520–560 nm) and mCherry (600–650 nm). The pinhole was set at one Airy unit.

FRET was detected and quantified by lifetime imaging (FLIM) using a (time-domain) time correlated single photon counting approach. The inverted laser scanning microscope Leica SP8-SMD equipped with a 440-nm pulsed laser (Picoquant GmbH, Germany) tuned at 40 MHz and single-photon counting electronics (PicoHarp 300) was used to excite the donor alone (LIMP-2-mTFP1) and the donor in the presence of acceptor (LIMP-2-mTFP1+MPR-mVenus). The emitted blue photons (donor only) passed through a 460–500-nm filter and were detected with a HyD detector (Leica Microsytems). The acquisition times ranged from 2 to 3 min and at least 150 photons per pixel were collected in all cases. A 2 × 2 spatial binning was applied in all images to increase the signal to noise. The acquired fluorescence decays coming from regions of interest comprising one whole cell were deconvoluted with the instrument response function and fitted by a Marquandt nonlinear least-square algorithm using Symphotime software (Picoquant) with one- or two-exponential theoretical models. If FRET occurred the mean lifetime^21^ was shortened and a two species model (an interacting fraction corresponding to a population that relaxes through FRET (*f*_D_) and a non-interacting fraction in which the donor lifetime remains undisturbed (1−*f*_D_)) was applied. The donor lifetime obtained from a single exponential fit from cells (∼2.65 ns) expressing the donor alone was used for the non-interacting fraction of the double exponential model in the corresponding co-transfected cell^21^,^47^,^48^.


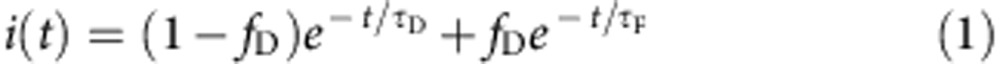


where *τ*_D_ is the fixed donor lifetime and *τ*_F_ is the discrete FRET lifetime. In this analysis, the total intensity (*I*_0_) was normalized to 1 and so that the pre-exponential factors lie in the range 0–1 (Fig. 8).

The true FRET efficiency (*E*) was calculated applying the formula:


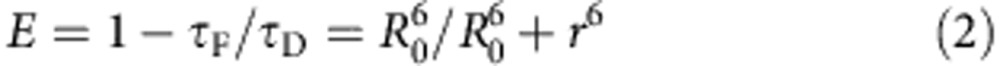


where *τ*_F_ is the FRET lifetime and *τ*_D_ is the donor lifetime; *R*_0_ is the Förster radius and *r* is the distance between the donor and the acceptor. The *R*_0_ for the mTFP1/Venus was calculated as in Padilla–Parra *et al*.^21^, and was 57 Å as reported in Ai *et al*.^49^ The average *E* was found to be 0.69±0.06 and the average distance between the two fluorescent proteins tagged to LIMP-2 and MPR was 49±2.5 Å. Note that the true FRET efficiency (*E*) is quite constant for all experiments (*n*=11), whereas *f*_D_ varied depending on the extent of the interaction (Fig. 8); this calculation highlights the importance of recovering *f*_D_.

The pixel by pixel *f*_D_ was obtained calculating the mean lifetime <*τ*> using the first 1,200 channels (20 ps per channel) of the fluorescence decay in a pixel by pixel manner, a binning of 2 × 2 was applied to increase the signal to noise, although the mean lifetime is a parameter that is quite robust with just ∼100 photons per pixel^50^.


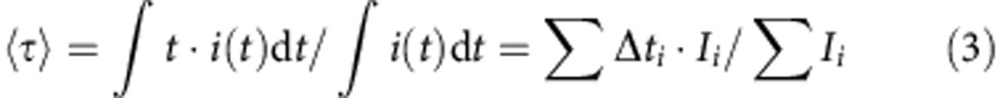


In order to obtain *f*_D_, these images were then treated with ImageJ (http://rsbweb.nih.gov/ij/) applying the formula:


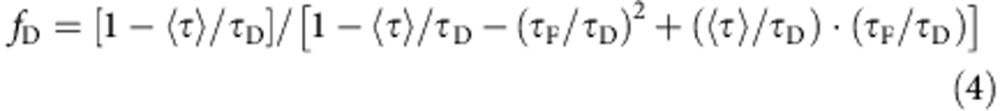


where <*τ*> is the mean lifetime, *τ*_D_ is the fixed donor lifetime and *τ*_F_ (∼0.83 ns) is the fixed discrete FRET lifetime obtained from fitting (see above). The *f*_D_ plots together with the statistics (*t*-tests) where calculated using Sigma Plot.

### Western blot analyses of secreted LIMP-2 luminal domain

Wild-type and mutant domains were cloned into a pNeoSec vector with a C-terminal His tag and expressed in HEK293T cells for 2 days. Protein samples (conditioned media (M) or cell lysate (C)) were reduced (5 mM DTT) and separated by SDS–PAGE gel. Molecular weight markers (BenchMark) were used (Invitrogen). Western blots were probed with anti-His tag (Penta His, Qiagen), followed by goat anti-mouse IgG–horseradish peroxidise conjugate (Sigma). The enzymatic activity of horseradish peroxidise was detected using the Amersham ECL Western blotting detection kit (GE Healthcare Life Sciences).

## Author contributions

All authors designed the research and wrote the paper; Y.Z., J.R. and S.P.-P. performed the experiments, and together with E.E.F. and D.I.S analysed the results.

## Additional information

**How to cite this article:** Zhao, Y. *et al*. Lysosome sorting of β-glucocerebrosidase by LIMP-2 is targeted by the mannose 6-phosphate receptor. *Nat. Commun.* 5:4321 doi: 10.1038/ncomms5321 (2014).

**Accession codes**: Atomic coordinates and structure factors for space group *C*222_1_ and *C*2 have been deposited with the Protein Data Bank under accession codes 4Q4B and 4Q4F, respectively.

## Supplementary Material

Supplementary Information Supplementary Figures 1-6, Supplementary Tables 1-2 and Supplementary References

## Figures and Tables

**Table 1:** Data collection and refinement statistics.

*Data collection*		
Space group	*C*222_1_	*C*2
*Cell dimensions*		
* a, b, c* (Å)	63.7, 95.4, 217.8	89.7, 63.4, 114.4
* *α, β, γ (°)	90, 90, 90	90, 102.3, 90
Resolution (Å)	50.0–2.80 (2.90–2.80)*	50.0–2.80 (2.90–2.80)
Unique reflections	15,620 (1,535)	15,244 (1,460)
*R*_merge_	0.180 (0.636)	0.119 (0.378)
*I*/σ*I*	9.0 (2.2)	7.1 (1.8)
Completeness (%)	92.2 (94.6)	96.2 (91.5)
Redundancy	6.6 (5.0)	2.5 (2.1)
*Refinement*		
Resolution (Å)	50.0–2.80	50.0–2.80
No. of reflections	13,289/770	13,688/763
*R*_work_/*R*_free_	0.222/0.283	0.223/0.269
No. of atoms		
* *Protein	3,128	3,148
* *Ligand/ion	363	378
B**-factors (Å^2^)		
* *Protein	45	53
* *Ligand/ion	57	70
* Root mean square deviations*		
* *Bond lengths (Å)	0.005	0.005
* *Bond angles (°)	1.2	1.3

^*^Highest resolution shell is shown in parenthesis.
