# Supplementary material for: Lysosome sorting of β-glucocerebrosidase by LIMP-2 is targeted by the mannose 6-phosphate receptor
Source: Nat Commun. 2014 Jul 14;5:4321. doi: 10.1038/ncomms5321 (PMC4104448; doi:10.1038/ncomms5321)
Supplement: Supplementary Information — Supplementary Figures 1-6, Supplementary Tables 1-2 and Supplementary References [file ncomms5321-s1.pdf]

a

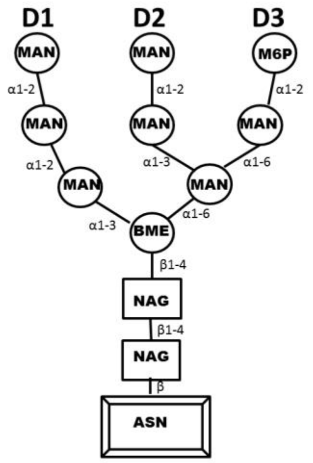

b

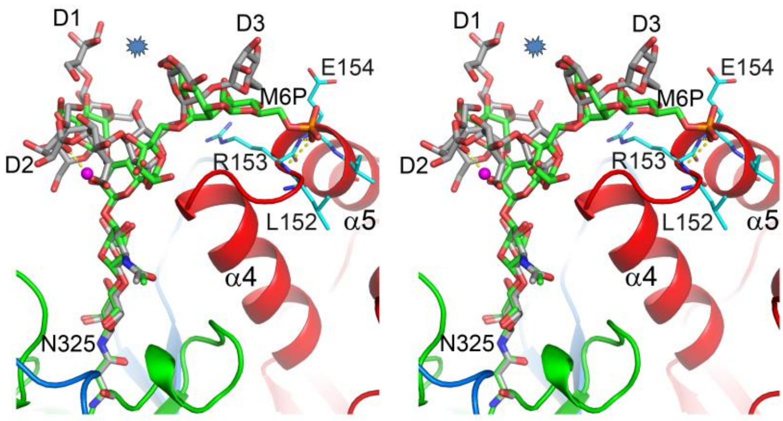

Supplementary Figure 1 | The N-linked P-Man$_9$GlcNAc$_2$ at N325. (**a**) Diagrammatic representation of the N-linked P-Man$_9$GlcNAc$_2$. The three branches of the oligosaccharide are designated D1, D2 and D3. NAG: N-Acetylglucosamine; BME: β-Mannose; MAN: α-Mannose; M6P: Mannose-6-phosphate. (**b**) Stereo diagram comparing the N-linked glycan at N325 in space groups *C222$_1$* (grey) and *C2* (green). Protein side-chains around M6P in space group *C2* are shown as cyan sticks. The star marks the approximate position of a helical turn (residues 121-125) from a symmetry related molecule.

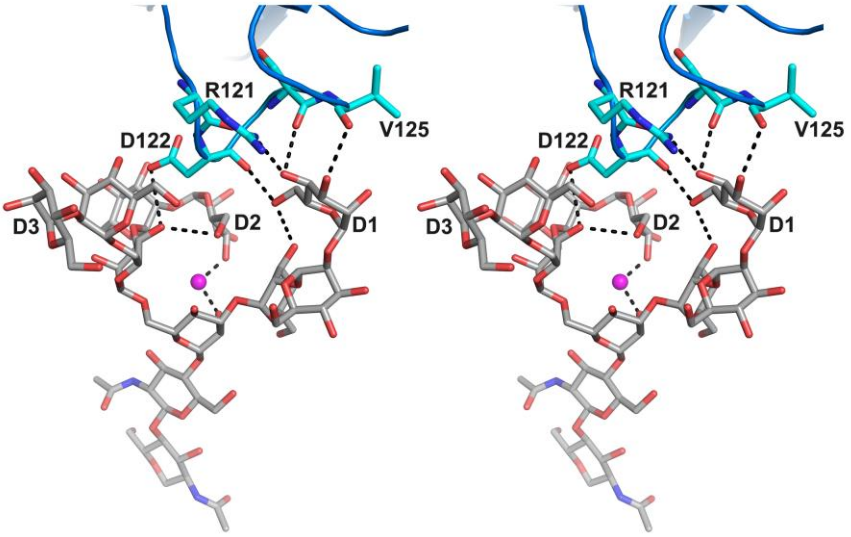

Supplementary Figure 2 | Stereo diagram showing interactions between the N-linked glycan at N325 and a helical turn (residues 121-125) from a symmetry related molecule in space group *C222₁*. The N-linked P-$Man_9GlcNAc_2$ is drawn as grey sticks, Na^+ as magenta sphere. The main-chain of the symmetry related molecule is shown as ribbons, residues interacting with the glycan as cyan sticks. The dotted lines represent H-bonds.

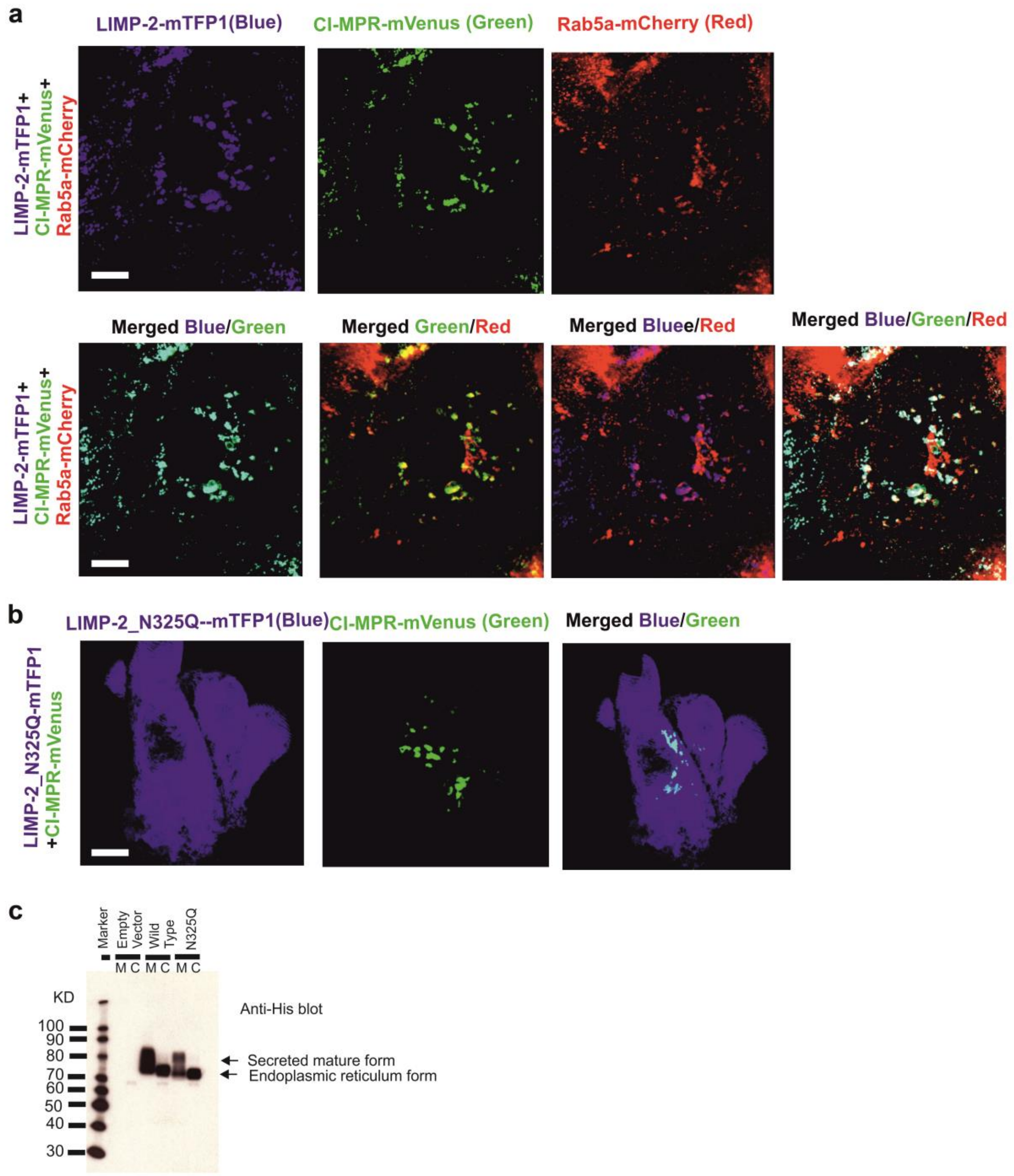

Supplementary Figure 3 | LIMP-2 and CI-MPR co-localization in living cells and Western blot analyses. (**a**) First row shows confocal images of COS-7 cells co-expressing LIMP2-mTFP1, MPR-mVenus and Rab5a-mCherry respectively. Second row shows overlapping images. It shows that LIMP-2 and CI-MPR are well co-localized, and both of them are only partially co-localized with the early endosome marker Rab5a. (**b**) The diffusive pattern of LIMP-2-N325Q-mTFP1 (first panel), the punctate appearance of CI-MPR-mVenus (second panel) and overlapping image shows little co-localization. The scale bar is 5 μm. (**c**) Western blot analyses of secreted wild type and mutant constructs of LIMP-2 luminal domain. Wild type and N325Q mutant LIMP-2 luminal domain with a C-terminal His tag were expressed in HEK293T cells for 2 days. Protein samples from the conditioned media (M) or cell lysate (C) were reduced and resolved on the SDS-PAGE gel, blotted to nitrocellular membrane and probed with anti-His tag antibody.

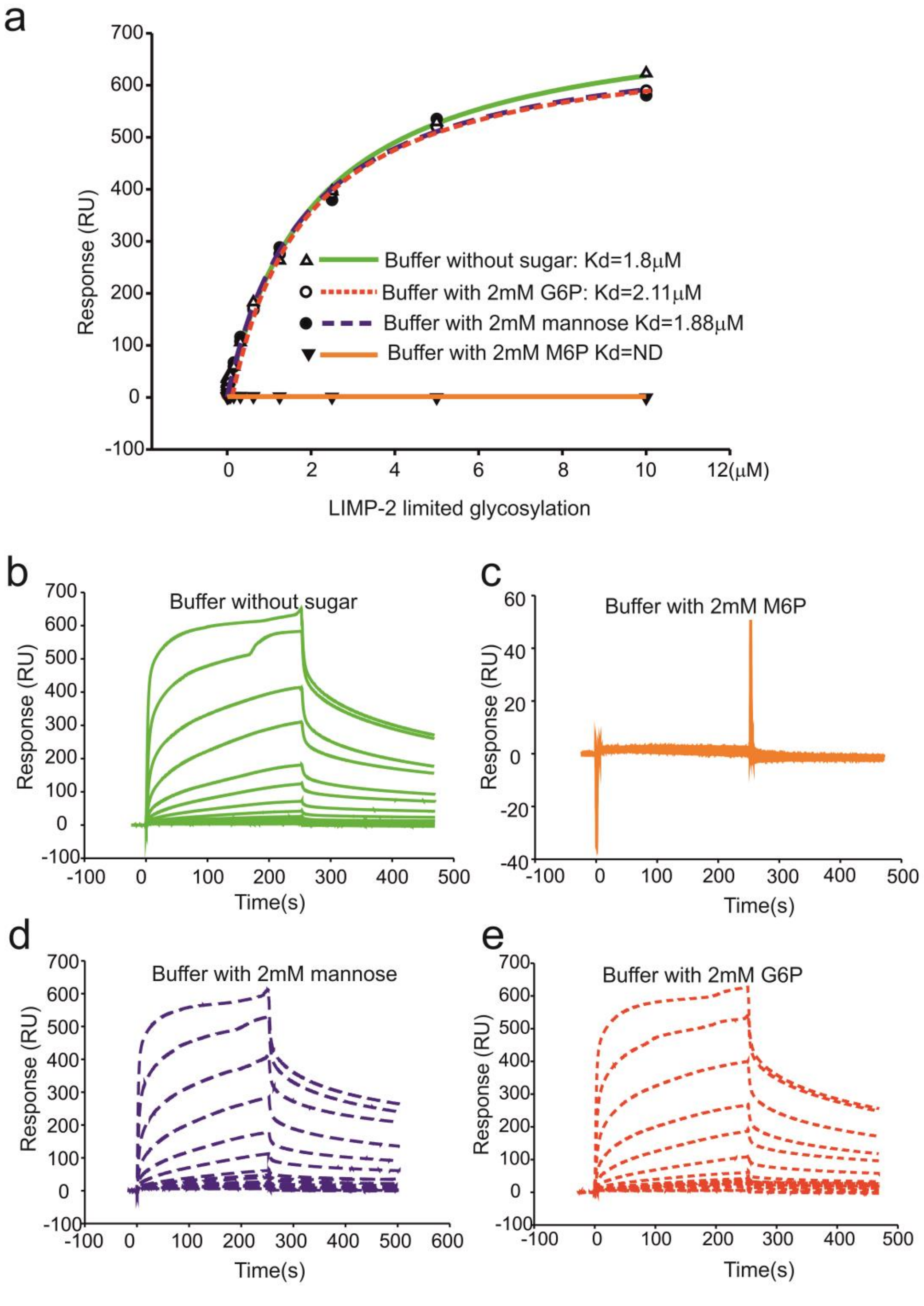

Supplementary Figure 4 | SPR experiments of **LIMP-2 (limited glycosylation) and CI-MPR binding**. Biotinylated human CI-MPR domains 1-15 was immobilized in a CM5 chip. LIMP-2 produced from HEK293S GNTI- cells (limited glycosylation) was used as analyte. (**a**) Summary of effects of sugars on MPR1-15 and LIMP-2 binding. (**b-e**) SPR sensorgrams of MPR1-15 and LIMP-2 binding without sugar, with 2mM M6P, 2mM mannose and 2mM G6P, respectively.

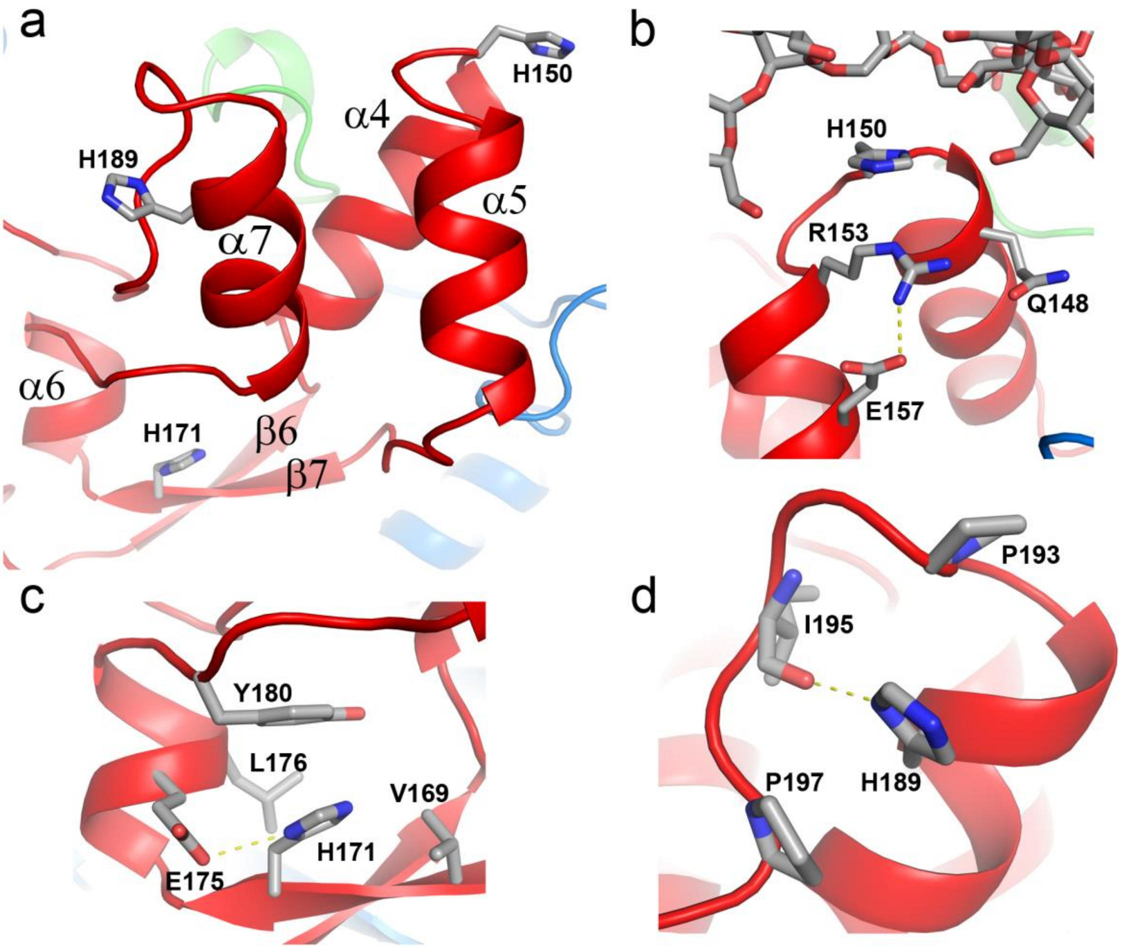

Supplementary Figure 5 | Overall structure of the LIMP-2 domain II. (**a**) Overall structure of domain II and the positions of three histidine residues within the domain. (**b**), (**c**) and (**d**) Environments around H150, H171 and H189, respectively. Yellow dotted lines represent H-bonds.

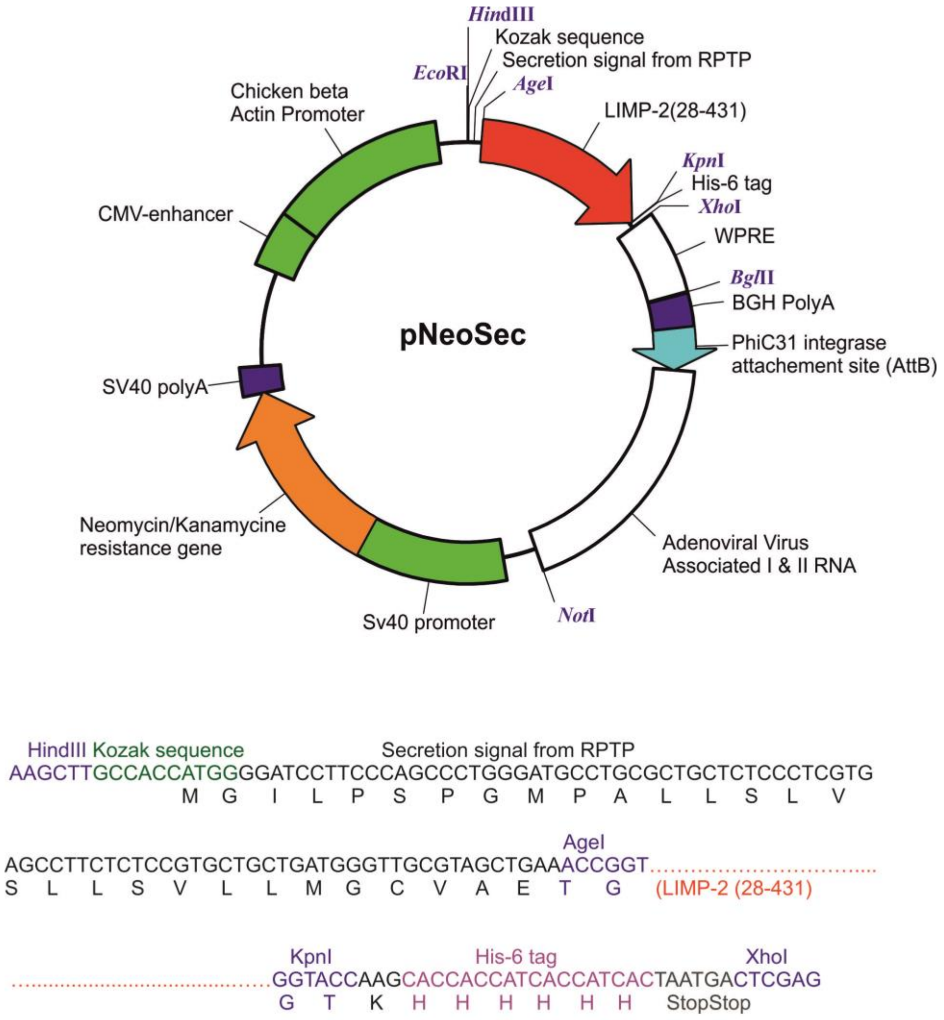

Supplementary Figure 6 | PneoSec-LIMP2 vector for stable cell line generation. The vector contains a phiC31 attachment site (AttB) (for site specific recombination with pseudo AttP sites within the mammalian cell genome when the PhiC31 integrase gene (pgk-phiC31/pCB92) is co-transfected) and a mammalian cell selection cassette (an SV40 promoter, a neomycin/G418 resistance gene and a polyadenylation (PolyA) sequence). The primary expression cassette contains the cytomegalovirus (CMV) enhancer, a chicken beta Actin promoter, a Kozak sequence, an optimised receptor tyrosine phosphatase (RPTP) sigma secretion signal from pHLSec, a Woodchuck Hepatitis virus posttranscriptional regulatory element (WPRE)[1], and a bovine growth hormone (BGH) poly A. An Adenovirus Virus-Associated RNA I & II sequence is also included for binding the double-stranded RNA-activated protein kinase, DAI[2] thus suppressing the double stranded RNA response. The vector is of kanamycin resistance for bacterial selection. The primary cassette cloning junction details are described below the vector map.

	N45	N68	N105	N206	N224	N249	N304	N325	N412
C2	1	5	0	6	1	3	2	7	5
C222$_1$	1	4	0	2	1	3	2	11	5

Supplementary Table 1 | Number of glycan residues modelled at each glycosylation site of LIMP-2 in space group *C2* and *C222*$_1$. Note: electron density at N105 site confirms the site is glycosylated, but is not sufficient for modelling the glycan.

**Primers for LIMP-2 protein expression**	LIMP-2-V28-F	gtacaccggtGTCTTCCAGAAGGCTGTAGAC
	LIMP-2-T431-R	cagtggtaccAGTGTTAATCATAGACTTCAGTC
**Primers for LIMP-2 FLIM-FRET experiments**	LIMP-2-M1-F	catgaattcaccATGGGCCGATGCTGCTTCTAC
	LIMP-2-T478-R	catcggtaccGGTTCGAATGAGGGGTGCTCT
	LIMP-2-N325Q-F	CTGGGCTCAGGAGTTCTGcagGTCAGCATCTGCAAGAATG
	LIMP-2-N325Q-R	CATTCTTGCAGATGCTGACctgCAGAACTCCTGAGCCCAG
	CI-MPR-Q41-F	gctcaccggtCAGGCCGCCCCGTTCCCCGAGCTGT
	CI-MPR-G2293-R	catccgtacgGTCGTCCCCGGGATTCTCGCTGT
**Primers for SPR experiments**	GBA-1-F	catgaattcgccaccATGGAGTTTTCAAGTCCTTCC
	GBA-1-R	cagtcgtacgCTGGCGATGCCACAGGTAGGTG
	CI-MPR-Q41-F	gctcaccggtCAGGCCGCCCCGTTCCCCGAGCTGT
	CI-MPR-L489R	catcggtaccCAGCGCGGACAGGTCATAGCG
	CI-MPR-I2491-R	catcggtaccGATGTGTAAGAGGTCCTCGTCGCTG
	LIMP-2-H150T-F	TAGAGTGGTCCCAGGTGacaTTCCTCAGGGAGATC
	LIMP-2-H150T-R	GATCTCCCTGAGGAAtgtCACCTGGGACCACTCTA

Supplementary Table 2 | Primers used for PCR amplification. The restriction sites are underlined.

References

1. Loeb, J.E., Cordier, W.S., Harris, M.E., Weitzman, M.D. & Hope, T.J. Enhanced expression of transgenes from adeno-associated virus vectors with the woodchuck hepatitis virus posttranscriptional regulatory element: implications for gene therapy. *Hum Gene Ther* 10, 2295-305 (1999).
2. Green, S.R. & Mathews, M.B. Two RNA-binding motifs in the double-stranded RNA-activated protein kinase, DAI. *Genes Dev* 6, 2478-90 (1992).
